# The ethics of neuromodulation for anorexia nervosa: a focus on rTMS

**DOI:** 10.1186/2050-2974-2-10

**Published:** 2014-04-01

**Authors:** Alina Coman, Finn Skårderud, Deborah L Reas, Bjørn M Hofmann

**Affiliations:** 1 Regional Eating Disorders Service (RASP), Division of Mental Health and Addiction, Oslo University Hospital-Ullevål, P.O. Box 4956 Nydalen, Oslo N-0424, Norway; 2 Centre for Medical Ethics, University of Oslo, P.O. Box 1130, Blindern, Oslo N-0318, Norway; 3 Institute of Special Needs Education, University of Oslo, Sognsveien 250, Oslo N-0863, Norway

## Abstract

**Objective:**

Recently there has been emerging clinical and research interest in the application of deep brain stimulation (DBS) and repetitive transcranial magnetic stimulation (rTMS) to the treatment of anorexia nervosa (AN). To our knowledge, few studies have discussed ethical aspects associated with the increased use of neuromodulation in AN, some of which are quite specific to AN, despite the rapid development and dissemination of these new technologies.

**Method:**

We provide a brief overview of three published rTMS studies for AN and discuss ethical issues involved in the use of neuromodulation for AN.

**Results:**

In contrast to neurosurgery or DBS, rTMS is a less invasive technique, with less associated risk, and thus has greater potential to become a more widespread augmentation or add-on therapy for AN. New therapeutic procedures are promising, yet they raise ethical questions regarding informed consent and patient selection. Illness-specific issues surrounding authenticity and autonomy are important to consider, ensuring an ethical approach to treatment for patients with AN.

**Discussion:**

We argue that ethical investigations for neuromodulation techniques are timely and important, and discussions should go beyond the immediate goals of patient safety, consent, and risk and benefit, to consider broader ethical concepts such as authenticity and autonomy.

## Review

Recently, the US National Institute of Mental Health (NIMH) has moved towards a conceptualization of AN as a disorder of neurocircuitry which can be elucidated with modern tools and technological advances, especially genomics and neuroimaging [1]. At present, the aetiology and pathophysiology of AN are not completely known, yet genetic and neuroimaging studies are revealing greater insight into the underlying neural correlates which might be involved in the aetiology of AN. To date, neuroimaging, especially fMRI studies, suggest alterations in the dorsolateral prefrontal cortex area (DLPFC), insular, parietal and anterior cingulated cortex, all of which are areas involved in emotional processing, processing of reward, and body perception [2,3]. Traditionally, study paradigms used in neuroimaging have focused on investigating response to food and body stimuli, and illness-specific brain activity patterns related to symptoms. As has been acknowledged, underweight status is an ever-present confounder in investigations of AN, and while some studies indicate the presence of pre-existing traits [4] other studies indicate the existence of a scar effect due to underweight status or malnutrition [5]. At present, knowledge about the relationship between brain activity and illness, as informed by neuroimaging technologies, is correlational, yet findings are typically used as a rationale for the application of neuromodulation techniques to the treatment of AN. Neuromodulation techniques include invasive methods such as neurosurgery or deep brain stimulation (DBS), or non-invasive brain stimulation (NIBS), including repetitive transcranial stimulation (rTMS). Neuroimaging is seen as a promising tool to improve the efficacy of these techniques, especially rTMS by helping identify an appropriate stimulation target, which is considered to be a current main limitation [6].

Data demonstrating efficacy of neuromodulation methods for treating psychiatric disorders in general is accumulating, with a growing consensus towards the application of neuromodulation such as rTMS in treating depression, for example [7]. The invasive nature of these interventions, especially neurosurgery and DBS, and the inherent ethical considerations render these forms of treatment a last-resort for a subgroup of treatment-resistant patients. Anorexia nervosa is associated with high mortality rates [8] and a subgroup of patients experiences a chronic outcome [9]. As such, neuromodulation techniques have been increasingly proposed for the treatment of severe and longstanding AN proven refractory to standard treatment [10]. Such neuromodulation techniques are often not stand-alone treatment but they are used as additional or augmentation treatment in combination with behavioural training or other forms of therapy [11].

Some studies have shown positive results and provide proof-of-concept for AN as a disorder of brain circuitry, yet the mechanisms underlying neuromodulation techniques are not completely known. For AN, the evidence base for rTMS and DBS remains limited. Lipsman et al. [12] offer a thorough historical overview of the literature on the frequency and effects of neurosurgery. The lack of understanding neurocircuitry and the lack of specificity in targets of stimulation has made some critics advocate for ‘neuromodesty’ [13] and emphasize the necessity to investigate ethical aspects associated with neuromodulation. History has shown that it is important to stay vigilant and prevent misuse of these techniques in order to avoid negative experiences and attitudes that could deprive patients of therapeutic help [6].

We feel that rapid advances and new research priorities have arguably introduced a paradigm shift in defining and treating AN, making a renewed discussion of associated ethical concerns timely and important [11,12,14]. Some have argued that elucidating important ethical concerns is vital, especially since new technologies are often approved and implemented without critical appraisal [15] and the promise of neuroscience might, for some, have a “seductive” allure [16]. This appears to be also the case for psychotropic medication that is still recommended to a group of patients with AN despite known adverse effects and lack of data supporting their efficacy [17].

Brain-based interventions, even neurosurgery for AN, are not new [18]. Some have argued that although techniques such as DBS or rTMS represent recent therapeutic advances, the relevant ethical challenges they pose have been previously addressed in the bioethical literature for other medical or psychological treatments [13]. However, whereas specific ethical issues related to DBS have been discussed more broadly for other psychiatric disorders [19-21], and recently for AN [22], few debates have focused on rTMS. Further, existing ethical evaluations of rTMS for other psychiatric disorders have focused predominantly on safety issues alone [6].

This article can be seen as a first step in the endeavor to shed light on specific ethical issues that the use of neuromodulation techniques pose for patients suffering from AN. Although some issues pertain to neuromodulation techniques at-large, we focus mainly on rTMS. Clinically speaking, we feel a focus on rTMS is timely and important, as this procedure is less invasive and might be applied less severe patients, thereby not constituting a ‘last-resort’ treatment, but possibly functioning as an additional or augmentation treatment which is more widely accessible to treatment providers. In contrast, DBS is a more invasive treatment with greater challenges to issues of safety, long-term effects and informed consent, and its use will most probably be guided by restrictive regulations.

### Neuromodulation techniques for AN: rTMS studies

Repetitive transcranial magnetic stimulation induces repetitive stimulation to cortical tissue with the help of magnetic field pulses from a magnetic coil placed outside the head. It either suppresses (at low frequency, 1 Hz) or excites (at higher frequency, 20 Hz) cortical activity, and its effect can propagate beyond the site of stimulation to a network of brain regions. The only Food and Drug Administration (FDA) approved therapeutic use of TMS is for stimulating the dorsolateral prefrontal cortex (DLPFC) for medication-resistant depression. However, the therapeutic efficacy data for depression is limited, partly because stimulation of DLPFC is based on a vague understanding of which specific mechanisms are stimulated, considering that DLPFC is connected with many other brain areas [23].

There are six studies that explore the effects of rTMS on bulimic disorders and findings are inconsistent, with some studies showing placebo effects while other reporting significant changes [10]. Three studies, of which one is a single case study, explore effects of rTMS for symptoms of AN. Table 1 provides a brief overview of clinical details and outcome for the three studies for AN. Initial findings are promising, yet mixed [24-26]. No studies have yet assessed the duration of improvement in symptoms beyond one month, or the longer-term and secondary effects of these interventions, and future studies will need to establish for which patients these interventions work best. Randomized controlled studies, which are underway in several countries including the UK and France, are necessary to delineate specific effects of rTMS and confirm the proof-of-concept for rTMS as a method which affects disturbed neural circuitry for reward and self-regulation [25]. Thus far, however, the use of these methods is exploratory and mostly confined to research settings and has not been routinely implemented as a therapeutic technique in the clinics.

**Table 1:** rTMS studies in anorexia nervosa

**Authors (year)**	**N**	**Patient characteristics**	**Treatment**	**Primary outcome**
Kamolz et al. (2008) [24]	1	Gender: female	Treatment of comorbid depression in a female patient with AN.	Improvement in depression was observed, as well as weight gain and fewer ED symptoms. After 1^st^ cycle, HAM-D scores decreased from 28 to 14. After 2^nd^ cycle, HAM-D scores decreased from 18 to 10. After 3^rd^ cycle, HAM-D scores decreased 18 to 11. During continuation therapy, HAM-D scores remained between 8 and 10.
		Age: 24 yrs.		
		BMI: 12.4	1^st^ cycle included 10 sessions within 16 days of high frequency rTMS to the left dorsolateral prefrontal cortex. 2^nd^ cycle included 6 sessions. 3^rd^ cycle included 10 sessions.	
		DOI: app. 4 yrs.		
			Continuation therapy included twice weekly rTMS sessions for 8 weeks.	BMI increased to approximately 16 kg/m^2^ after 12 weeks of rTMS.
Van den Eynde et al. (2013) [25]	10	Gender: female	Delivered 1 session of high frequency rTMS to the left dorsolateral prefrontal cortex.	1 patient dropped out prematurely due to discomfort.
		Mean age: 25 yrs.		
				On the visual analogue scale, significant reductions in feeling fat, feeling full, and anxiety, with a non-significant trend for decreased urge to exercise. No significant changes in mood, tension, hunger, “urge to eat” or “urge to restrict”. No reduction in cortisol levels, but found to be cardiac safe, as measured by blood pressure and heart rate.
			ED-related experiences were measured pre-post following exposure to visual and food stimuli	
		(18-44)		
		Mean BMI: 15.7		
		(13.8-17.8)		
		DOI: 10 yrs (3–30)		
McClelland et al. (2013) [26]	2	Patient A	Delivered 19–20 sessions of high frequency rTMS, applied to the left dorsolateral prefrontal cortex.	Patient A
		Gender: female		No change in weight at post-treatment or at 1-month follow-up. EDE-Q scores (except Eating Concern) were significantly lower at post-treatment and improvements were maintained at FUP. Some improvement in depression was observed.
		Age: 23 yrs.		
		BMI: 15.7	Within-session changes in ED-related experiences were measured. BMI, ED symptoms, and depression measured at pre-treatment, post-treatment, and 1-month follow-up.	
		DOI: 12 yrs.		
		Patient B		
		Gender: female		Patient B
				No change in weight at post-treatment or 1-month follow-up. Improvements in EDE-Q scores and depression were observed.
		Age: 52		
		BMI: 16.4		
		DOI: 35 yrs.		

*Note:* rTMS = repetitive transcranial magnetic stimulation; BMI = body mass index (kg/m^2^); DOI = duration of illness; HAM-D = Hamilton Depression Scale; EDE-Q = Eating Disorder Examination-Questionnaire, FUP = follow-up period.

### Ethical issues in neuromodulation for AN

In the treatment of AN, ethical discussions have mostly focused on clinical issues related to underweight, malnutrition, and the capacity to refuse treatment. However, with the increased use of brain interventions for treating AN, other ethical concerns specific to the application of (mechanical) brain interventions need elaboration.

Ethical assessment of the use of neuromodulation is often based on four principles: respect for autonomy, beneficence, non-maleficence, and justice. Table 2 provides an overview of the application of these ethical principles to the case of rTMS in AN. The following section summarizes and relates these aspects to principalism, as this is the most influential approach in bioethics.

**Table 2:** rTMS in AN and relevant ethical considerations

**Ethical principle**	**Threats pertinent for rTMS in AN**
Non-maleficence (do no harm)	Risks
	Side-effects and long-term effects
Beneficence (do well)	Effectiveness
	Patient selection
Respect for autonomy	Informed consent
	(Emotional and cognitive) capacity issue due to underweight
	Comorbidity, mortality, resistance to treatment
Justice	Patient selection, Resource allocation, Research deprivation

#### Balancing non-maleficence and beneficence

So far, the risks associated with participating in rTMS are low; however, since there is no evidence for the long-term effects and efficacy of rTMS, the risk-benefit ratio is difficult to assess [21]. Hence, utilitarian analysis fails to provide clear guidance in decision-making, and the beneficence is unclear. A pilot study for rTMS showed reductions in feeling of fatness and fullness, and anxiety, but fewer changes in mood, tension or urge to exercise [25]. In the future, it will also be relevant to establish criteria for evaluating effectiveness. If rTMS is proven effective and alleviates symptoms, it is relevant to establish the criteria for patient inclusion that should be adopted before neuromodulation is recommended, and establish the patient group for whom rTMS is relevant. In Van den Eynde’s study [25], the group of participants investigated was heterogeneous, and included individuals with different duration of disorders, which make it hard to know whether neuromodulatory interventions are more effective for less severe patients or chronic patients. Difficulties and disagreement over the definition of severity, e.g., in terms of BMI-alone, level of impairment, or other eating-related or general psychopathology, has thus far rendered the assessment of beneficence complicated.

Related to this argument, illness duration and refractoriness to treatment is often invoked as a rationale for neuromodulation [12]. Some have argued that moderate or a less severe illness might in fact be more responsive to brain interventions [27]. In AN, chronicity is typically seen after a period of 10 years [28]. Treating only patients with a long duration of illness may do injustice to individuals with a shorter duration of illness [27]. Thus ensuring a favorable risk-benefit ratio has also a moral aspect where issues of safety have to be balanced with the desire to help a group of patients who are not helped by other methods. This poses problems for autonomy and informed consent.

#### Respect for autonomy

Respect for autonomy is usually ensured by proper informed consent. At present, rTMS (and DBS) interventions for AN are often a last–resort treatment for patients who do not respond to other treatments. It has often been underlined in the ethics literature that patients participating in such last-resort exploratory research or treatment trials are “desperate” for a solution. As such, this subgroup of treatment refractory patients may be “overly motivated’ to participate in exploratory research and to expose themselves to risks [13]. This poses challenges to the capacity of these patients to fully understand the nature of intervention and implications of participation. Depression can affect perception of risks associated with participating in research [19], although some argue that depression in itself is not enough to compromise judgment [29]. For AN, the condition of severe underweight poses some additional ethical considerations of competency and non-maleficence. Severe underweight can lead to involuntary hospitalization and forced feeding based on the assumption that individuals lack cognitive or emotional capacity to make decisions [30]. Offering neuromodulation treatment to patients who are emaciated or malnourished poses inherent ethical dilemmas given the potential incapacity associated with underweight status.

While some rTMS studies conducted so far have excluded severely underweight patients, protecting them from harm, other studies have included patients with a BMI from 12.3.

Chronicity of illness, underweight and willingness to participate in last resort interventions, be it proven or experimental, can raise questions about the validity of informed consent. The validity of a consent coming from a person that is severely underweight, refractory to treatment, yet willing to undergo brain interventions, can be questioned. Assessing the validity of such consent will be dependent on objective and established criteria for competence. However, it is also dependant on the kind of policy adopted, either a restrictive, paternalistic attitude or a permissive one “permitting people to make unencumbered decision about how to govern their own lives”, and on how low the competence bar is set [13]. In cases where the competence bar is set low, it is essential to disclose to the participant all known and unknown information about the intervention in order to ensure informed consent.

The “overtly motivated,” possibly depressed patient is a general ethical concern. However, in AN, a lack of motivation and reluctance to engage in treatment has more often been the case. Especially pharmacological treatment, for example, antipsychotic medication such as olanzapine, is associated with higher drop-out than cognitive [31] and cognitive-behavioural treatment [32]. Further, the drop-out rate for adults with AN is higher (40%) than for adolescents in family therapy, in which the drop-out rates fall around 10-20% [32]. However, reluctance to medication might be explained by fears of weight gain, which can be a secondary effect of olanzapine [33]. Although it remains yet unknown how patients view neuromodulatory treatments, rTMS has been shown to reduce feelings of fullness or fatness, and to lessen anxiety, which may have greater appeal. In this case, the concern is rather, when the patient is motivated, how to proceed ethically. Given the current limited status of knowledge of neurocorrelates for AN, of the efficacy of mechanisms for brain stimulation and long-term effects, critics have drawn attention to the fact that neuromodulation means little. To proceed ethically would mean an invitation to “hyper disclosure” [13].

Another threat to autonomous participation in neuromodulatory interventions is the risk for *therapeutic misconception*, adopting the belief that participating in research will alleviate suffering. Informed consent should therefore aim to avoid misunderstanding, and facilitate autonomy, by making explicit the current experimental nature of the research, and ensure that the patient is explicitly informed about what rTMS can offer and not offer.

It is also possible that investigator bias, or simply the desire to understand the relation between the brain and illness, or to alleviate intense suffering, leads to overstatements of the current state of knowledge and this can impede cautious proceeding [13]. Especially in light of the high mortality and morbidity rate, AN may challenge the therapists’ clinical judgment and bias them to recommend the available new treatments, despite an evidence base which is still evolving. On the other hand, any negative attitudes or bias against brain interventions may encourage therapists to withhold treatment from patients [6].

#### Justice

Critics have drawn attention to the need to balance ethical concerns and (over) protection of rights that has sometimes led to “research deprivation” [34]. This is perhaps especially applicable to children and adolescents with AN, with a consideration of young individuals’ agency [35] and the need to proceed cautiously.

Researchers have already pointed to the perplexities of providing sound scientific evidence for medicated treatment and how consideration for safety by including the adult population entails high drop-out and biases samples [32]. This has made researchers inclined to advocate intensification of interventions at early stages of the disorder instead of conducting more randomized studies [32]. Others have warranted a more detailed and critical re-evaluation of the meaning and criteria for resistance to treatment [36], as it is an often-invoked argument for use of neuromodulation techniques but little described in the studies [12].

#### Autonomy and beyond: authenticity

Whether such interventions are proven effective in the future and the medical risks low, it will still be necessary to provide adequate care and attention to the individual and also longer-term follow-up. Resistance to standard treatment is often invoked by patients on the grounds of threats to authenticity, where aspects of AN are seen as integrative of the self and therefore the patient is less willing to change [37]. All treatments, medical or psychological, can be said to change aspects of the self, and to either threaten or contribute to the development of what is conceived as “authentic”, although this concept is contested [38]. Although the understanding of authenticity and the role of this concept in treatment is still poorly understood, studies show that threats to authenticity are experienced by patients although mostly in the beginning of the treatment as grounds to refuse treatment [37]. Although concerns for authenticity appear to be amenable to change over time, and should not just be taken at face-value as a valid reason to respect patients’ refusal of treatment, an ethical approach to treatment requires nevertheless a consideration of patients’ concerns with authenticity.

Technological assisted treatment, as with rTMS, touches on broader debates about the nature and meaning of symptoms and neuromodulatory techniques. As mentioned earlier, the results from early trials of rTMS seem to show reductions in feelings of fullness and fatness. Yet patients may be reluctant to address ideas regarding overconcern of shape and weight, which may be intertwined with the subjective meaning of the illness ascribed by the patient [39]. For example, feelings of fatness have been shown to be related to expressing emotions of sadness or disgust that are displaced on the body, and depend on context [40]. Preoccupation with the body, restriction, purging can be seen as concrete representation of psychological states, a tendency some have termed ‘psychological equivalence’ [41].

Importantly, differentiating between the meaning of symptoms according to the patient and the aetiology of symptoms demands clinical skills from the therapists. It is feasible that recommending a neuromodulation technique might alienate the patient by rendering important and ego-syntonic symptoms reducible to defect neuro-networks. Earlier studies show that although biological framing of the disorder can reduce self-blame, it can also disturb self-understanding and the perception of volition seen as significant for recovery [42,43]. Therefore the therapist is in a position to address participants concerns about authenticity and agency when they jointly determine treatment goals [37].

#### A complementary criterion: individual experience, cultural and social aspects

The risk-benefit calculations must be based on assessing effectiveness both at the group level but also at the individual level [44]. At present, it remains uncertain how patients might experience these technologies and whether they present threats to authenticity. We argue that this should be done not only based on quantitative measures of benefit, but should be complemented by a first-person account of the role such technologies play in the life of the sufferer. For example, such first-person accounts have been shown to be a contribution to the debate about medication of children with ADHD [38]. The answers to these questions could ensure a complete ethical evaluation and ethical approach to the use of neurotechnologies, and prevent “clinical push” and technological enthusiasm which may create stigma that would deprive sufferers of therapeutic benefit [6].

Lastly, ethical perspectives may be influenced by cultural and societal values. While the media is increasingly presenting the brain as a “capital that should be optimized” [45], and the brain becomes an integrated, although not dominant part of our self-understanding [43,46], the public may become more cautious about brain interventions as neuromodulation techniques have a controversial history [6]. Such social and cultural values might also be impacted by the specific features of the disorder, and attention to context is warranted [13]. Limited knowledge exists regarding factors, which might impede or encourage acceptability of neuromodulation interventions for both sufferers, health professionals and the general public [14].

## Conclusions

This article can be seen as a first step in the attempt to shed light on specific ethical issues that the use of neuromodulation techniques pose for patients with AN. Although promising, findings are mixed and future studies are warranted to demonstrate their efficacy. The risks for rTMS are currently low and thus further experimental trials can proceed and ensure consistent evidence for the efficacy of the neuromodulation techniques. However, considering the current limited knowledge, careful attention to context and practice should follow. Ethical issues such as careful informed consent and clinical sensitivity have to be taken into account when gaining this knowledge and applying these techniques. Further, if these methods prove effective, it is nevertheless relevant that patients, families, caregivers, treatment providers, and the public-at-large are involved in discussions regarding potential ethical dilemmas.

In this article, we have applied a principle-based approach as described by Beauchamp and Childress [47]. Other perspectives exist and could have been followed; for example, a deontological approach would have highlighted human worth and dignity. However, our approach addressed concerns for authenticity and meaning without reference to intention-based ethical theories. The utilitarian perspective has been covered by the principle of beneficence. Various types of utilitarianism exist, but they are all faced with challenges when the knowledge on risks and benefits is vague, uncertain, or absent. Correspondingly, procedural approaches, such as discourse ethics [48] and “accountability for reasonableness” [49,50] are relevant, especially when assessing health care interventions under uncertainty. However, they may face challenges in AN as the persons themselves may refuse to be participants in the discourse and deliberation.

In conclusion, we argue that investigations of neurostimulation techniques such as rTMS in AN go beyond establishing efficacy to a broader phenomenological, first-person understanding of how technologies affect users’ lives. As such, future studies and ethical debates about the efficacy and acceptability of these methods should therefore investigate attitudes both among health providers and patients. Finally, considering that research on neurobiology of AN will most likely continue to rapidly develop in concert with NIMH research priorities, concomitant ethical debates should follow in parallel with such developments.

## Competing interests

The authors declare that they have no competing interests.

## Authors’ contributions

AC drafted the manuscript, completed the literature search and analysis of the material. DR contributed to developing the manuscript, presentation of review material, as well as revising for intellectual content and language editing. FS and BH contributed to validation and developing of ideas, and were involved in approval of final draft of the paper. All authors read and approved the final manuscript.

## References

[B1] Insel T Mental illness Defined as Disruption in Neural Circuits 2011 [updated 2011National Institute of Mental Health]; Available from: http://www.nimh.nih.gov/about/director/2011/mental-illness-defined-as-disruption-in-neural-circuits.shtml

[B2] Pietrini F Castellini G Ricca V Polito C Pupi A Faravelli C Functional neuroimaging in anorexia nervosa: a clinical approach Eur Psychiatry 2011 26 3 176 182 10.1016/j.eurpsy.2010.07.011 20934859

[B3] Uher R Brammer MJ Murphy T Campbell IC Ng VW Williams SCR Treasure J Recovery and chronicity in anorexia nervosa: brain activity associated with differential outcomes Biol Psychiatry 2003 54 9 934 942 10.1016/S0006-3223(03)00172-0 14573322

[B4] Kaye WH Wagner A Fudge JL Paulus M Adan RAH, Kaye WH Neurocircuity of eating disorders Behavioral Neurobiology of Eating Disorders 2011 6 Heidelberg: Springer Berlin 37 57 10.1007/7854_2010_85 PMC5957512 21243469

[B5] Pruis TA Keel PK Janowsky JS Recovery from anorexia nervosa includes neural compensation for negative body image Int J Eat Dis 2012 45 8 919 931 10.1002/eat.22034 22729811

[B6] Horvath JC Perez JM Forrow L Fregni F Pascual-Leone A Transcranial magnetic stimulation: a historical evaluation and future prognosis of therapeutically relevant ethical concerns J Med Ethics 2011 37 3 137 143 10.1136/jme.2010.039966 21106996

[B7] Slotema CW Dirk Blom J Hoek HW Sommer IEC Should we expand the toolbox of psychiatric treatment methods to include repetitive transcranial magnetic stimulation (rTMS)? a meta-analysis of the efficacy of rTMS in psychiatric disorders J Clin Psychiatry 2010 71 7 873 10.4088/JCP.08m04872gre 20361902

[B8] Arcelus J Mitchell AJ Wales J Nielsen S Mortality rates in patients with anorexia nervosa and other eating disorders: a meta-analysis of 36 studies Arch Gen Psychiatry 2011 68 7 724 10.1001/archgenpsychiatry.2011.74 21727255

[B9] Steinhausen HC The outcome of anorexia nervosa in the 20th century Am J Psychiatry 2002 159 8 1284 1293 10.1176/appi.ajp.159.8.1284 12153817

[B10] McClelland J Bozhilova N Campbell I Schmidt U A systematic review of the effects of neuromodulation on eating and body weight: evidence from human and animal studies Eur Eat Disord Rev 2013 21 6 436 455 10.1002/erv.2256 24155246

[B11] Kadosh RC Levy N O’Shea J Shea N Savulescu J The neuroethics of non-invasive brain stimulation Curr Biol 2012 22 4 R108 R111 10.1016/j.cub.2012.01.013 22361141 PMC4347660

[B12] Lipsman N Woodside DB Giacobbe P Lozano AM Neurosurgical treatment of anorexia nervosa: review of the literature from leucotomy to deep brain stimulation Eur Eat Disord Rev 2013 21 6 428 435 10.1002/erv.2246 23873668

[B13] Morse SJ New therapies, old problems, or a plea for neuromodesty AJOB Neuroscience 2012 3 1 60 64 10.1080/21507740.2011.635632

[B14] Mendelsohn D Lipsman N Bernstein M Neurosurgeons’ perspectives on psychosurgery and neuroenhancement: a qualitative study at one center J Neurosurg 2010 113 6 1212 1218 10.3171/2010.5.JNS091896 20524827

[B15] Jeanne L Shannon B Why the FDA can’t protect the public BMJ 2010 341 7780 966 968

[B16] Weisberg DS Keil FC Goodstein J Rawson E Gray JR The seductive allure of neuroscience explanations J Cogn Neurosci 2007 20 3 470 477 18004955 10.1162/jocn.2008.20040 PMC2778755

[B17] Fazeli PK Calder GL Miller KK Misra M Lawson EA Meenaghan E Lee H Herzog D Klibanski A Psychotropic medication use in anorexia nervosa between 1997 and 2009 Int J Eat Disord 2012 45 8 970 976 10.1002/eat.22037 22733643 PMC3726215

[B18] Sifneos PE A case of anorexia nervosa treated succesfully by leucotomy Am J Psychiatry 1952 109 5 356 360 12985994 10.1176/ajp.109.5.356

[B19] Fisher CE Dunn LB Christopher PP Holtzheimer PE Leykin Y Mayberg HS Lisanby SH Appelbaum PS The ethics of research on deep brain stimulation for depression: decisional capacity and therapeutic misconception Ann NY Acad Sci 2012 1265 1 69 79 10.1111/j.1749-6632.2012.06596.x 22812719 PMC3624886

[B20] Glannon W Stimulating brains, altering minds J Med Ethics 2009 35 5 289 292 10.1136/jme.2008.027789 19407032

[B21] Pisapia JM Halpern CH Muller UJ Vinai P Wolf JA Whiting DM Wadden TA Baltuch GH Caplan AL Ethical considerations in deep brain stimulation for the treatment of addiction and overeating associated with obesity AJOB Neuroscience 2013 4 2 35 46 10.1080/21507740.2013.770420 PMC5687095 29152408

[B22] Wu H Van Dyck-Lippens PJ Santegoeds R van Kuyck K Gabriels L Lin G Deep-brain stimulation for anorexia nervosa World Neurosurg 2013 80 3 S29 S 22743198 10.1016/j.wneu.2012.06.039

[B23] Fox MD Buckner RL White MP Greicius MD Pascual-Leone A Efficacy of transcranial magnetic stimulation targets for depression is related to intrinsic functional connectivity with the subgenual cingulate Biol Psychiatry 2012 72 7 595 603 10.1016/j.biopsych.2012.04.028 22658708 PMC4120275

[B24] Kamolz S Richter D-PMM Schmidtke A Fallgatter AJ Transkranielle magnetstimulation gegen komorbide depression bei anorexie Nervenarzt 2008 79 9 1071 1073 10.1007/s00115-008-2537-8 18661116

[B25] Van den Eynde F Guillaume S Broadbent H Campbell IC Schmidt U Repetitive transcranial magnetic stimulation in anorexia nervosa: a pilot study Eur Psychiatry 2013 28 2 98 101 10.1016/j.eurpsy.2011.06.002 21880470

[B26] McClelland J Bozhilova N Nestler S Campbell IC Jacob S Johnson-Sabine E Schmidt U Improvements in symptoms following neuronavigated repetitive transcranial magnetic stimulation (rTMS) in severe and enduring anorexia nervosa: findings from two case studies Eur Eat Disord Rev 2013 21 6 500 506 10.1002/erv.2266 24155247

[B27] Kuhn J Gaebel W Klosterkoetter J Woopen C Deep brain stimulation as a new therapeutic approach in therapy-resistant mental disorders: ethical aspects of investigational treatment Eur Arch Psychiatr Clin Neurosci 2009 259 2 135 141 10.1007/s00406-009-0055-8 19876671

[B28] Strober M Freeman R Morrell W The long-term course of severe anorexia nervosa in adolescents: survival analysis of recovery, relapse, and outcome predictors over 10ΓÇô15 years in a prospective study Int J Eat Dis 1997 22 4 339 360 10.1002/(SICI)1098-108X(199712)22:4<339::AID-EAT1>3.0.CO;2-N 9356884

[B29] Appelbaum PS Assessment of patients’ competence to consent to treatment N Engl J Med 2007 357 18 1834 1840 10.1056/NEJMcp074045 17978292

[B30] Tan J Hope T Widdershoven G, McMillan J, Hope T, Scheer LS Treatment refusal in anorexia nervosa: a challenge to current concepts of capacity Empirical Ethics in Psychiatry 2008 Oxford: Oxford University Press 187 210

[B31] Dahlgren CL Lask B Landrø N-I Rø O Developing and evaluating cognitive remediation therapy (CRT) for adolescents with anorexia nervosa: a feasibility study Clin Child Psychol Psychiatry 2013 1359104513489980 10.1177/1359104513489980 23761592

[B32] Halmi K The perplexities of conducting randomized, double-blind, placebo-controlled treatment trials in anorexia nervosa patients Am J Psychiatr 2008 165 10 1227 1228 10.1176/appi.ajp.2008.08060957 18829874

[B33] Bissada H Tasca G Barber AM Bradwejn J Olanzapine in the treatment of low body weight and obsessive thinking in women with anorexia nervosa: a randomized, double-blind, placebo-controlled trial Am J Psychiatr 2008 165 10 1281 1288 10.1176/appi.ajp.2008.07121900 18558642

[B34] Arnold LE Stoff DM Cook E Jr Cohen DJ Kruesi M Wright C Hattab J Graham P Zametkin A Castellanos FX Ethical issues in biological psychiatric research with children and adolescents J Am Acad Child Adolesc Psychiatry 1995 34 7 929 939 10.1097/00004583-199507000-00017 7649964

[B35] Singh I Victimology versus character: new perspectives on the use of stimulant drugs in children J Med Ethics 2013 39 6 372 373 10.1136/medethics-2012-101283

[B36] Abbate-Daga G Amianto F Delsedime N De-Bacco C Fassino S Resistance to treatment and change in anorexia nervosa: a clinical overview BMC psychiatry 2013 13 1 294 10.1186/1471-244X-13-294 24199620 PMC3879222

[B37] Hope T Tan J Stewart A Fitzpatrick R Anorexia nervosa and the language of authenticity Hastings Cent Rep 2011 41 6 19 29 22238901 10.1002/j.1552-146x.2011.tb00153.x

[B38] Singh I Not robots: children’s perspectives on authenticity, moral agency and stimulant drug treatments J Med Ethics 2013 39 6 359 366 10.1136/medethics-2011-100224 22930677 PMC3664392

[B39] Habermas T On the uses of history in psychiatry: diagnostic implications for anorexia nervosa Int J Eat Dis 2005 38 2 167 182 10.1002/eat.20159 16134113

[B40] Espeset EMS Nordbø RHS Gulliksen KS Skarderud F Geller J Holte A The concept of body image disturbance in anorexia nervosa: an empirical inquiry utilizing patients’ subjective experiences Eat Disord 2011 19 2 175 193 10.1080/10640266.2011.551635 21360367

[B41] Skårderud F Sommerfeldt B Fonagy P Den reflekterende kroppen. Mentalisering og spiseforstyrrelser Nord J of Child Adol Psychother 2012 2 6 21

[B42] Easter MM Not all my fault: genetics, stigma, and personal responsibility for women with eating disorders Social Sci Med 2012 75 8 1408 1416 10.1016/j.socscimed.2012.05.042 PMC3495131 22819736

[B43] Coman A Skårderud F Hofmann BM A disorder of a vulnerable self: anorexia nervosa patients’ understanding of disorder and self in the context of fMRI brain scanning EHPP 2013 15 2 120 134

[B44] Schermer M Ethical issues in deep brain stimulation Frontiers in Integrative Neuroscience 2011 5 17 10.3389/fnint.2011.00017 PMC3096836 21625629

[B45] O’Connor C Rees G Joffe H Neuroscience in the public sphere Neuron 2012 74 2 220 226 10.1016/j.neuron.2012.04.004 22542177

[B46] Pickersgill M Cunningham-burley S Martin P Constituting neurologic subjects: neuroscience, subjectivity and the mundane significance of the brain Subjectivity 2011 4 346 365 10.1057/sub.2011.10

[B47] Beauchamp TL Childress JF Principles of bioethical ethics 2013 New York: Oxford University Press

[B48] Habermas J Moral Consciousness and Communicative Ethics Cambridge 1990 Cambridge: MIT Press

[B49] Daniels N Mechanic D, Rogut L, Colby D, Knickman J Accountability for reasonable limits to care Policy Challenges in Modern Health Care 2005 New Brunswick: NJ: Rutgers University Press 228 248

[B50] Daniels N Sabin JE Accountability for reasonableness: an update BMJ 2008 337 228 248 10.1136/bmj.a1850 18845595

